# Small towns limit dispersal and reduce genetic diversity in populations of Texas horned lizards

**DOI:** 10.1002/ece3.70112

**Published:** 2024-08-06

**Authors:** Ashley E. Wall, Daniella Biffi, Alexis Ackel, Raymond W. Moody, Tom K. Stevens, Dean A. Williams

**Affiliations:** ^1^ Department of Biology Texas Christian University Fort Worth Texas USA; ^2^ Andrews Institute of Mathematics & Science Education Texas Christian University Fort Worth Texas USA; ^3^ USAF 72nd ABW/CE, Environmental Compliance Natural Resources Tinker AFB Oklahoma USA

**Keywords:** effective population size, isolation, microsatellites, mitochondrial DNA, urban ecology, urbanization

## Abstract

There is a general expectation that urban populations will be fragmented and the movement of individuals will be restricted leading to low effective population sizes, low genetic diversity, higher inbreeding, and higher differentiation than populations living in more continuous habitat. In this study, we compare the genetic diversity and differentiation of Texas horned lizards that are found in four small towns (Kenedy, Karnes City, Rockdale, and Smithville) in Texas and at Tinker Air Force Base, Oklahoma to populations that occur in 16 natural areas and to an introduced population in South Carolina. We also present more detailed spatial genetic data and home range data for several of the towns. Texas horned lizards (*Phrynosoma cornutum*) living in small towns have lower genetic diversity, higher differentiation, and smaller effective population sizes than populations located in more natural areas. There was evidence for human‐mediated movement of lizards into town; however, it has not been enough to counteract the effects of drift. Home range size is smaller in town than in more natural areas. Genetic patterns suggest dispersal occurs over short distances and is inhibited across areas with a high percent of impervious surface and major roads. These data suggest that effective planning to maintain suitable habitat and corridors to facilitate movement is critical to maintaining small terrestrial species like the Texas horned lizard and must be integrated into the early stages of urban development.

## Graphical abstract

Urban populations typically experience fragmentation and restricted movement of individuals, resulting in lower genetic diversity, increased inbreeding, and higher genetic differentiation compared to populations in continuous habitats. Texas horned lizards in small towns have lower genetic diversity and higher differentiation compared to populations in natural areas, likely due to restricted dispersal influenced by impervious surfaces such as buildings and roads. Our results suggest that even low levels of urbanization can have negative effects on small terrestrial vertebrates and so effective planning for maintaining suitable habitat and corridors to facilitate movement must be integrated into the early stages of urban development.

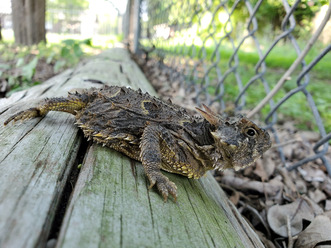

## 1 INTRODUCTION

Urbanization results in habitat degradation and fragmentation (Czech et al., 2000; Hamer & McDonnell, 2010; McKinney, 2006). Physical structures, exotic species, and vegetative homogenization can inhibit movement of urban residents and negatively affect the biotic requirements of individuals (Fusco et al., 2020; Riley et al., 2006; Shochat et al., 2010; Tucker et al., 2018). Consequently, there is a general expectation that urban populations will be fragmented, and the movement of individuals will be restricted leading to low effective population sizes, low genetic diversity, higher inbreeding, and higher differentiation than populations living in more continuous habitat (Miles et al., 2019; Schmidt et al., 2020). Other patterns have been found, however, such as an increase in the effective size and genetic diversity of some species because they are human commensals, or their movement is facilitated by human activity (Carlen & Munshi‐South, 2021). There may also be no measurable effect on the movement and genetic structure of some highly mobile species like birds or bats since they can easily move across the urban environment (Richardson et al., 2021; Schmidt et al., 2020).

Reptiles living within the urban matrix have been studied much less than birds and mammals (French et al., 2018). Reptiles are generally thought to have lower dispersal ability than mammals and birds and therefore may be more negatively impacted by urbanization (Putman & Tippie, 2020). The few studies that have been conducted on the genetic structure of reptiles living within the urban matrix have found contrasting signals. Delaney et al. (2010) compared three lizard species in Los Angeles, California, USA and found that the presence of one highway produced large genetic differences between populations of the same species on either side of the highway, indicating that it had essentially eliminated movement of these species. Eastern water dragons (*Intellagama lesueurii*) living in four city parks in Brisbane, Australia have similar genetic diversity, but significant differentiation compared to rural populations (Littleford‐Colquhoun et al., 2017). Other studies of lizards have found little or no effect of urbanization on levels of genetic diversity and differentiation, possibly because not enough time has passed for previously large populations to experience detectable drift or because the species is well adapted to living in urban areas (Beninde et al., 2016; Krawiec et al., 2015). More population genetic studies of reptiles living within the urban matrix need to be conducted across a range of urban development to better understand the variability in species responses to urbanization.

The Texas horned lizard (*Phrynosoma cornutum*) was once abundant throughout Texas, with its native range encompassing much of Texas and Oklahoma, parts of Kansas, Arizona, New Mexico, Louisiana, and northern Mexico (Price, 1990). The species has declined through much of its range, including a virtual disappearance from eastern Texas, and so is considered threatened in Texas and a species of concern in Oklahoma (Donaldson et al., 1994; Henke, 2003; Price, 1990). The loss of suitable habitat due to urbanization and agriculture is probably in large part responsible for these declines, although there are also other interrelated factors including the introduction of red imported fire ants (*Solenopsis invicta*), and the decline of harvester ants (*Pogonomyrmex* spp.), which are a major food source for Texas horned lizards (Donaldson et al., 1994).

A recent meta‐analysis suggests that lizards in the family Phrynosomatidae are less negatively affected by human‐modified habitat than other groups of lizards, possibly because many of them live in arid habitats with sparse vegetation that may mimic disturbed sites (Doherty et al., 2020). Consistent with this pattern, Texas horned lizards have remained a wildlife component of some small towns (<10,000 people) in Texas, although they have disappeared from larger urban areas. Texas horned lizards are widely loved by people and so have been moved through the pet trade and by residents introducing them on their property (Williams et al., 2019). Therefore, it is possible that towns could facilitate the anthropogenic movement of this species, and so lizards in towns may have higher or similar genetic diversity to lizards living in more natural areas. The species' anatomy, reliance on crypsis, and generally sedentary lifestyle, however, suggests they have low dispersal potential and so are vulnerable to becoming isolated in urban areas by roads, buildings, and walls (Sherbrooke, 2003; Williams et al., 2019). Most studies on the effects of urbanization on biodiversity and genetic structure have been conducted in large cities (>100,000 people) and it is unclear to what extent patterns observed in highly urbanized areas can be applied to smaller cities or towns (Łopucki & Kitowski, 2017). Large urban areas have more potential barriers to dispersal and so populations might be expected to exhibit the effects of isolation more strongly than in small towns (Norton et al., 2016). Understanding the effects of small towns on the movement and genetic structure of native wildlife is especially important for areas like Texas in which 99% of cities have less than 100,000 people and 35% have below 10,000 people (*n* = 5662 cities ranging in size from 100 – 2.3 million people) (https://www.texas‐demographics.com/cities_by_population, accessed June 25, 2023).

In this study, we use nuclear microsatellite loci and the mitochondrial control region to compare the genetic diversity and differentiation of Texas horned lizards in five small towns in Texas and Oklahoma to populations that occur in 16 natural areas in Texas, Colorado, and New Mexico reported in Williams et al. (2019) and to an introduced population in South Carolina, USA (Heuring et al., 2019). We predicted that town populations would be effectively isolated from surrounding populations due to this species' low dispersal capacity. Town populations were therefore predicted to have low genetic diversity, small effective population sizes, and higher differentiation compared to populations in natural areas. We then present home range size and configuration in the towns of Karnes City and Kenedy and more detailed spatial genetic data for the population in Karnes City. We predicted that roads, buildings, and other constructed structures would impede dispersal and constrain home range size of this species resulting in small home ranges, evidence for short distance dispersal, and lower genetic similarity between individuals separated by major roads and development.

## 2 METHODS

### 2.1 Study sites

**FIGURE 1:**
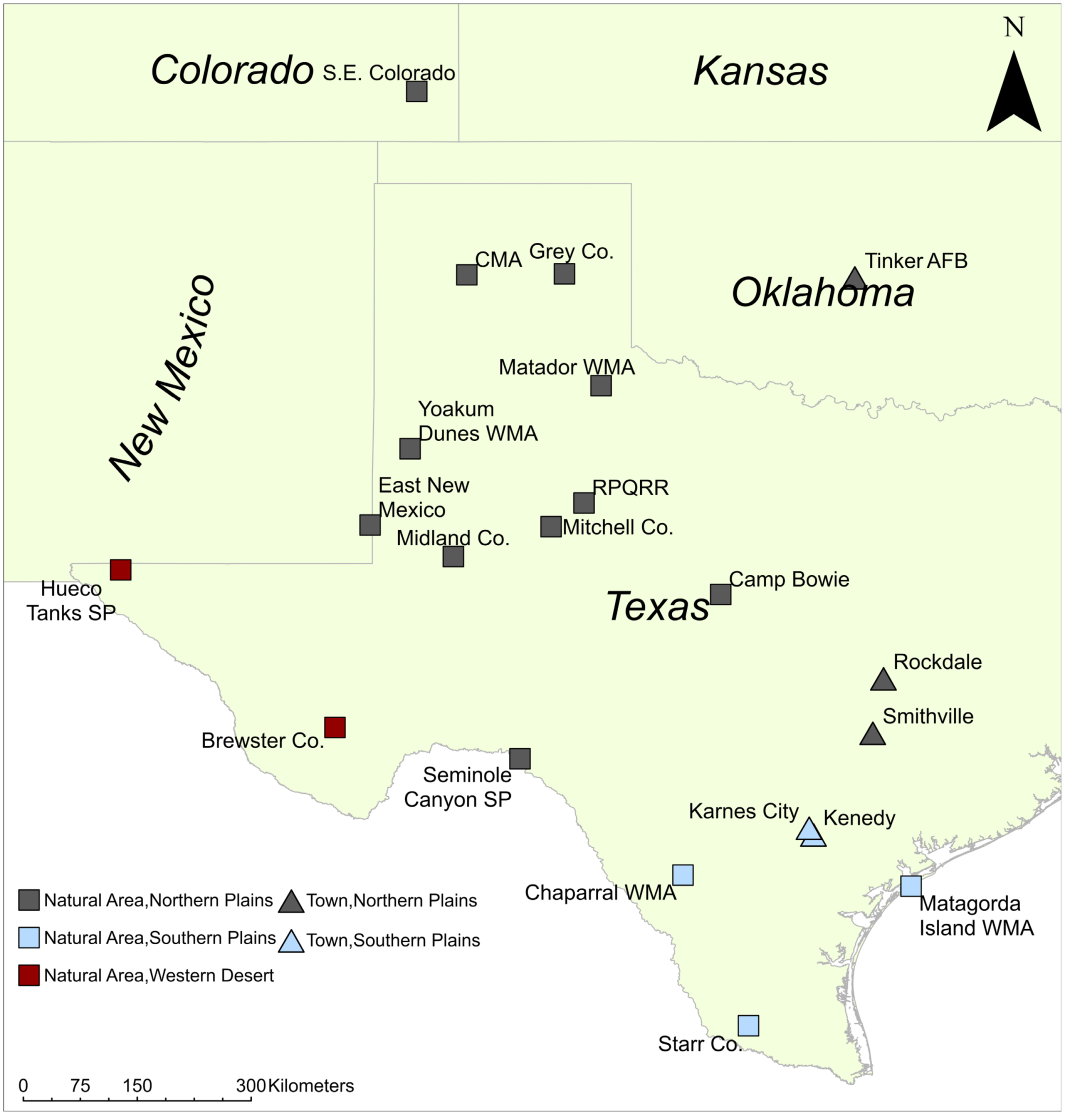
Sampling locations for Texas horned lizards (*Phrynosoma cornutum*) in towns and natural areas (from Williams et al., 2019). Colors indicate which of the three genetic clusters (Western Deserts, Northern Plains, Southern Plains) individuals in a location belong to (see text).

We collected 189 Texas horned lizard tissue samples from four small towns (Smithville, Rockdale, Kenedy, and Karnes City—population size 3042–5851 people) in Texas and from Tinker Air Force Base (TAFB) in Midwest City, Oklahoma (57,000 people) in 2013 using cloacal swabs or toe clips (Williams et al., 2012) (Figure 1). Tinker Air Force Base has been the focus of a long‐term population study and has been described in several publications (Endriss et al., 2007; Wolf et al., 2013). This site is an isolated 15 ha natural site that is surrounded by housing and industrial developments. Smithville and Rockdale still have populations of lizards but are in a region of Texas where the lizards have declined and disappeared over the past several decades in surrounding areas (Donaldson et al., 1994). The relative isolation of lizards in these two towns has led to speculation that these lizards may have been introduced by residents. In Smithville, lizards were only found in a northeast neighborhood of town and in Rockdale lizards were found throughout town but at very low densities. Kenedy and Karnes City are in south Texas where horned lizards still occur in areas surrounding the towns (Wall, 2014, Williams, unpub. data). Kenedy and Karnes City have been the focus of long‐term study into the coexistence of horned lizards and human‐modified habitats (Ackel, 2016; Alenius, 2018; Mirkin et al., 2021; Tucker et al., 2023; Wall, 2014). In Karnes City, the lizards are found throughout town but in Kenedy they are now confined to a single area in the north of town. Within towns, the lizards are found in parks, school yards, abandoned lots, alleyways, and residents' yards that contain a mix of native grasses and forbs, scattered trees, shrubs, and bare patches of ground.

Texas horned lizard phylogeographic structure consists of a strong split at mitochondrial DNA that occurs between the western deserts and the eastern plains (Finger et al., 2021; Williams et al., 2019). Nuclear loci also detect this split and further divide the eastern plains region into the northern plains and southern plains (Finger et al., 2021; Williams et al., 2019). Tinker Air Force Base is geographically located within the northern plains, and Kenedy and Karnes City are in the southern plains. Smithville and Rockdale are in a region of Texas where it is not clear which genetic cluster (northern or southern plains) they may belong since horned lizards declined and disappeared from this area before studies of genetic structure were conducted.

We compared the towns to 16 protected and rural areas in Texas, New Mexico, and Colorado, that were previously published in an extensive genetic study of Texas horned lizards (Williams et al., 2019) and to a published data set from an introduced population in South Carolina, USA (Heuring et al., 2019) (Figure 1). The 16 sites include 15 mainland sites and a site located on a barrier island (Matagorda Island WMA). Lizards were introduced into South Carolina sometime between 1920 and 1940, and they now occur on several barrier islands along the coast (Heuring et al., 2019).

### 2.2 Genetic analyses

DNA was extracted from cloacal swabs or toe clips preserved in Longmire's lysis buffer (100 mM Tris pH 8.0, 100 mM EDTA, 10 mM NaCl, 0.5% sodium dodecyl sulfate; Longmire et al., 1988) using a standard Proteinase K/ammonium acetate/isopropanol precipitation method as in Williams et al. (2012).

### 2.3 Genetic diversity and differentiation

We genotyped all 189 individuals at 10 previously used microsatellite loci and scored the genotypes using GeneMapper 5.0 (Life Technologies) as described in Williams et al. (2012, 2019). We used GenAlEx v6.5 (Peakall & Smouse, 2006, 2012) to calculate the number of alleles, allelic richness, observed (H_O_) and expected heterozygosity (H_E_), F_IS_, F_ST_, and standardized F'_ST_ (Meirmans & Hedrick, 2011). We calculated allelic richness using HP‐RARE 1.0 (Kalinowski, 2005). We tested for Hardy–Weinberg and genotypic linkage equilibrium using GENEPOP v4.2 (Rousset, 2008). We used permutation tests implemented in FSTAT 2.9.4 (Goudet, 2003) to test for differences in allelic richness, observed heterozygosity and F_ST_ between the five towns and 15 natural mainland sites.

We amplified and sequenced all 189 samples at a 353 bp section of the mitochondrial control region as in Williams et al. (2019). Polymerase chain reactions (PCR) were cleaned enzymatically with *ExoI* and *rSAP* using the manufacturer's protocols (New England Biolabs Ipswich). Products were sequenced in both directions using PCR primers and BrightDye® Terminator Cycle Sequencing Kit (MCLAB, South San Francisco) and electrophoresed on an ABI 3130XL Genetic Analyzer. Sequences were trimmed, edited, and put into contigs using Sequencher 4.8 (Gene Codes Corporation). One hundred and eight‐eight samples of 189 were sequenced successfully. Unique sequences have been deposited in GenBank under accession numbers OR843053‐OR843059. We used GenAlEx v6.5 to estimate mitochondrial haplotype diversity (*h*) and ɸPT for both the mitochondrial locus and for the microsatellite genotypes to compare population differentiation at the two marker types.

### 2.4 Bottlenecks and effective population size

We used the program BOTTLENECK (Cornuet & Luikart, 1996; Piry et al., 1999) to test for the genetic signature of a recent reduction in the effective population size (Ne) in the towns using the microsatellite genotypes. As recommended by Piry et al. (1999), heterozygosity at equilibrium was calculated using the two‐phase mutation model (TPM) with a probability of 95% for single‐step mutations and 5% multi‐step mutations since this model is believed to better approximate mutations at microsatellite loci than a pure stepwise mutation model (Di Renzo et al., 1994). A Wilcoxon sign‐rank test was then used to determine if a significant number of loci exhibited excess heterozygosity compared to the expectation at equilibrium.

We used NeEstimator v2.1 (Do et al., 2014) to calculate Ne for the towns and 16 natural areas using the LD method (Hill, 1981; Waples, 2006; Waples & Do, 2010). We used random mating and an allele frequency cut‐off of 0.05 when <25 individuals were sampled and a cut‐off of 0.02 when >25 individuals were sampled when calculating Ne (Waples & Do, 2010). We report the jackknife confidence intervals around Ne.

### 2.5 Anthropogenic movement of Texas horned lizards

We used STRUCTURE 2.3.4 (Pritchard et al., 2000) and the USEPOPINFO model to assign individuals in towns to the three major genetic clusters (northern plains, southern plains, and western deserts) found in Williams et al. (2019) to determine if the towns had ancestry that was consistent with their geographic location. If towns contain a mix of ancestry from two or three of these genetic clusters it would suggest that people had probably introduced some lizards into the towns from a different genetic cluster. We used samples from Williams et al. (2019) that were previously assigned to a cluster with ≥0.90 ancestry as learning samples and ran the Markov Chain Monte Carlo (MCMC) for F1,000,000 iterations following a burn‐in period of 100,000 iterations for *K* = 3 using the correlated allele frequencies model and assuming admixture for 10 runs. We then used CLUMPP 1.1.1 (Jakobsson & Rosenberg, 2007) to average q values across the 10 runs.

### 2.6 Home range use in Kenedy and Karnes City

During the 2013 summer active season, we fit individuals with A1065 beaded transmitters (1.4 g) (ATS—Advanced Telemetry Systems) in the towns of Kenedy and Karnes City to determine how the lizards utilized space within these towns. Transmitters were attached to the upper dorsal side using non‐toxic Mega Pro bonding glue (JB Cosmetics) and further secured with a collar made with fishing line covered with IV tubing. Individuals were released at the location of capture. In most cases, the collar prevented detachment of the transmitter from the individual following shedding events. In these cases, we reattached the transmitter dorsally. Using 14 transmitters, we tagged a total of 19 lizards (ten lizards in Karnes City (5 females, 5 males), six in Kenedy (4 females, 2 males)).

We located lizards daily from June through September 2013 using an R‐1000 telemetry receiver (Communications Specialists, Inc.) with a Yagi directional antenna (RA‐150) and recorded the GPS coordinates of each visually confirmed location. We located lizards at different times each day (from 8:00 to 12:00, 12:00 to 16:00, or 16:00 to 20:00) to reduce any bias that might result from lizards preferring to be in a certain part of their home range during a particular time of day.

Home ranges were constructed using the 95% and 100% Minimum Convex Polygon (MCP) methods calculated using ArcMET 10.1.11 in ArcGIS Desktop version 10.1. We calculated MCP areas for the 11 individuals that had ≥17 location points (i.e., 17 or more days of tracking) since home range area appeared to level off after this threshold. This value is similar to that used in other studies of horned lizards (Burrow et al., 2002; Endriss et al., 2007; Wolf et al., 2013).

### 2.7 Karnes City spatial genetic structure

Texas horned lizards are very cryptic and difficult to find, and so we used an expanded data set of 177 unique adult lizards (>69 mm snout‐to‐vent length (SVL)) collected between 2013 and 2015, for spatial genetic analyses using the microsatellite genotypes. Locations used for all lizards were at their initial point of capture.

**FIGURE 2:**
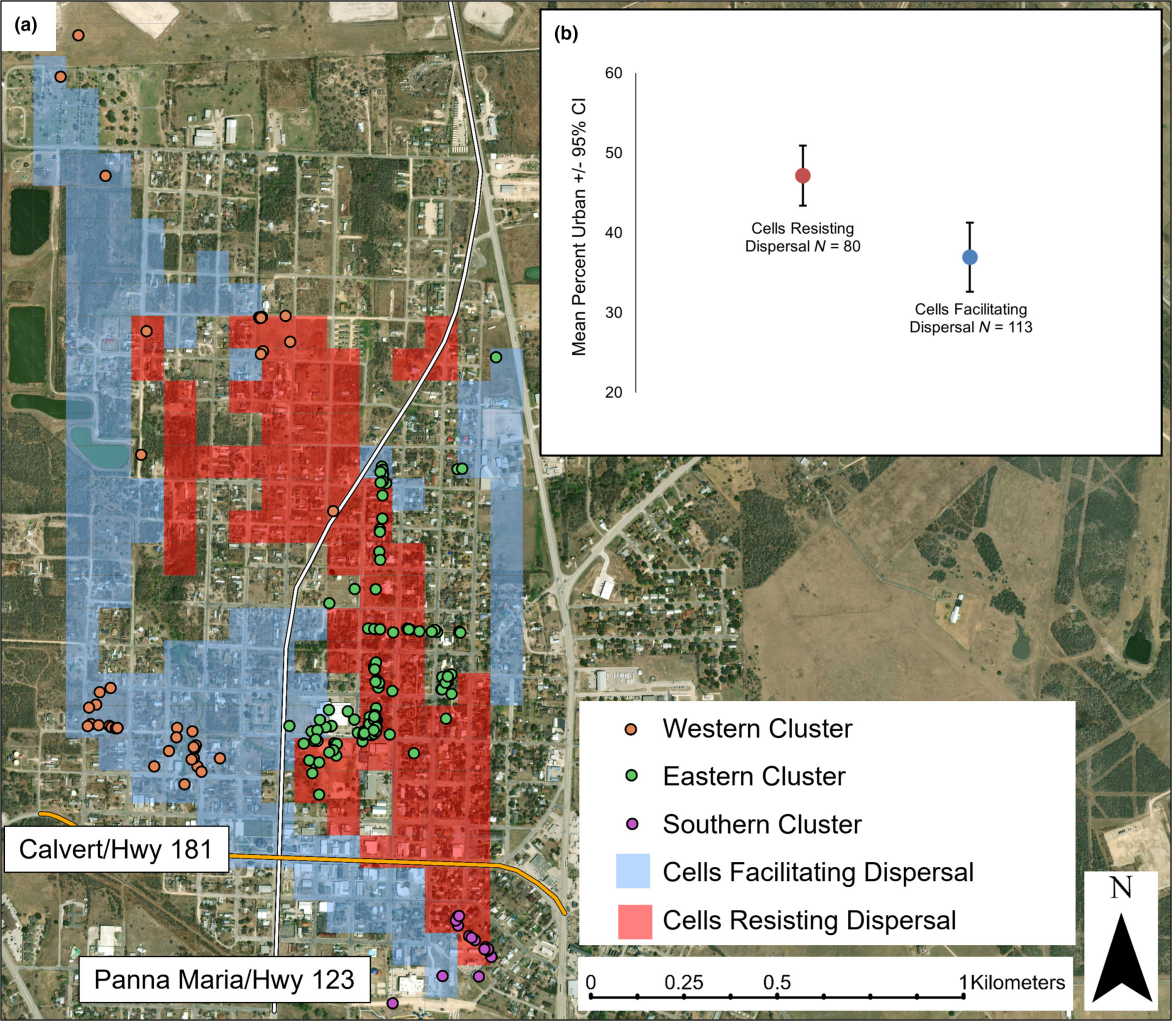
(a) The location of all 177 unique individual Texas horned lizards (*Phrynosoma cornutum*) in Karnes City and locations in the landscape that resisted and facilitated dispersal generated by ResDisMapper in Karnes City, TX, USA. The southern cluster of individuals occurred south of Calvert Avenue/Hwy 181. The eastern cluster occurred north of Calvert Avenue/Hwy 181 and east of Panna Maria Avenue/Hwy 123 and the western cluster occurred west of Panna Maria Avenue/Hwy 123. (b) Cells that resisted dispersal were more urban (more impervious surface) than cells that facilitated dispersal.

We calculated F'_ST_ across the two major two‐lane roads that intersect the town. The two roads are Calvert Avenue/Hwy 181 (speed limit 56 km/hr), (yearly mean traffic volume 3829, TxDOT TPP Statewide Annual Average Daily Traffic, 2013–2015) running from east to west and Panna Maria Avenue/Hwy 123 (speed limit 64 km/hr), (yearly mean traffic volume 1998, TxDOT TPP Statewide Annual Average Daily Traffic, 2013–2015) running north to south. Individuals were categorized as occurring south of Calvert Avenue/Hwy 181 (Karnes City South, *n* = 16 individuals), or north of Calvert Avenue/Hwy 181, which were then split into east of Panna Maria Avenue/Hwy 123 (Karnes City East, *n* = 119 individuals) or west of Panna Maria Avenue/Hwy 123 (Karnes City West, *n* = 42 individuals) (Figure 2a). We used STRUCTURE 2.3.4 to cluster individuals in Karnes City using the LOCPRIOR models developed by Hubisz et al. (2009) for situations with weak population structure. We ran the Monte Carlo Markov Chain (MCMC) for 1,000,000 iterations following a burn‐in period of 100,000 iterations for *K* = 1–4 using the correlated allele frequencies model and assuming admixture for 10 runs. STRUCTURE can give misleading results both for the number of populations and individual ancestry if there is uneven sampling across clusters (*K*) (Puechmaille, 2016; Wang, 2017). We used the recommendations of Wang (2017) and set the prior for admixture to allow ALPHA to vary between clusters and we decreased the initial ALPHA from 1.0 to 0.2. The most likely K was estimated using the method of Evanno et al. (2005). We used CLUMPP 1.1.1 (Jakobsson & Rosenberg, 2007) to average q values across the 10 runs for the most likely K.

We used spatial genetic autocorrelation methods to ask if there was evidence for short‐distance dispersal within Karnes City (Peakall et al., 2003; Smouse & Peakall, 1999). In GenAlEx v6.5, we ran a combined analysis for each sub‐population (East, West, and South) using variable distance classes that maximized sample sizes for the larger distance classes (Peakall et al., 2003). Significance was determined using 1000 permutations of the data and 1000 bootstraps of the individual correlation coefficients. The genetic correlation (*r*) is significant for a distance class if it falls outside the 95% confidence limits and if the bootstrap values do not include zero.

We used Resistance to Dispersal Mapper (ResDisMapper; Tang et al., 2020) to explore the impacts of the two main roads and the degree of impervious surface on dispersal. ResDisMapper maps resistance to dispersal at small spatial scales without requiring prior knowledge of the impact of environmental features on dispersal. ResDisMapper uses a four‐step process to map resistance to dispersal: (1) calculates genetic and geographic distances for all possible pairs of individuals to generate isolation by distance (IBD) values, and then fits the IBD trend to generate residuals for all pairs of individuals; (2) visualizes the distribution of IBD residuals as line segments between individuals across the landscape; (3) creates a grid with a user defined cell size and uses IBD residual values from all line segments that intersect a grid cell to calculate that cell's resistance (mean of the IBD residual values), statistical certainty of resistance, and statistical significance of resistance (positive resistance indicates a location that resists dispersal, whereas negative resistance indicates a location that facilitates dispersal); and (4) visualizes the resistance map. We used the absolute genetic distance (Prevosti et al., 1975) to calculate IBD values and generated residuals by fitting a linear trend to IBD values. We created a grid with a cell size of 1 ha and used all IBD residuals to calculate resistance (infinite geographic distance range). We used 95% CI to determine statistical certainty of resistance values in each grid cell, where certainty is achieved when the CIs do not overlap zero.

We linked resistance to dispersal values from ResDisMapper to urbanization in the landscape using two methods. First, we used ArcGIS Pro v. 2.2.0 to map grid cells from ResDisMapper where certainty for either facilitating or resisting dispersal was met, and then calculated the amount of impervious surface within each grid cell using data from the USGS National Land Cover Database (Dewitz, 2021) for 2014. We then compared the amount of urbanization in cells that resisted dispersal to cells that facilitated dispersal. To explore the impact of roads on gene flow, we looked for significant resistance values in groups of line segments generated in step 2 of the ResDisMapper process that crossed only one of the two major highways (Calvert Avenue/Hwy 181 and Panna Maria Avenue/Hwy 123). Lines that crossed both highways or no highways were excluded.

## 3 RESULTS

### 3.1 Genetic diversity and differentiation

Across the 189 samples, there were six individuals that were missing one locus in their genotypes (0.3% missing loci). In the expanded dataset of 177 individuals from Karnes City, there were 11 individuals missing one (8 individuals) or two loci (3 individuals) (1% missing loci). Smithville had a single locus (*Pc09*) with a significant heterozygote deficit while none of the other loci and towns had significant heterozygote deficits or excess. Overall loci, Rockdale had a significant heterozygote deficit (*p* = .0001) and Kenedy had a significant heterozygote excess (*p* = .001) (Table 1). There were 3 out of 225 pairwise locus comparisons that were significantly out of genotypic linkage equilibrium (*p* < .0002). All three pairs of loci were unique and occurred once in three of the towns (Smithville, TAFB, and Karnes City).

**TABLE 1:** Mean ± SE genetic diversity measures for Texas horned lizards, *Phrynosoma cornutum*, in Texas towns and Tinker Air Force Base, Oklahoma. N is the number of individuals genotyped at both microsatellite loci and the mitochondrial control region; Na is the average number of alleles; H_O_ is observed heterozygosity; H_E_ is expected heterozygosity; and F_IS_ is the inbreeding coefficient at 10 microsatellite loci.

Town	*N*	Na	H_O_	H_E_	F_IS_	Nh	h
Smithville	47	8.3 ± 0.58	0.79 ± 0.03	0.81 ± 0.01	0.02 ± 0.04	4	0.46
Rockdale	13	6.4 ± 0.52	0.58 ± 0.04	0.72 ± 0.02	0.16 ± 0.04*	6	0.76
Tinker AFB	37	8.6 ± 0.92	0.84 ± 0.03	0.80 ± 0.02	−0.06 ± 0.02	4	0.66
Karnes City	75	9.0 ± 1.19	0.77 ± 0.02	0.79 ± 0.02	0.03 ± 0.02	1	0.00
Kenedy	17	5.5 ± 0.63	0.68 ± 0.07	0.61 ± 0.06	−0.15 ± 0.06*	3	0.52

*Note*: Nh is the number of mitochondrial haplotypes at the control region and h is haplotype diversity.

*Significant deviation from 0 (*p* < .001).

**FIGURE 3:**
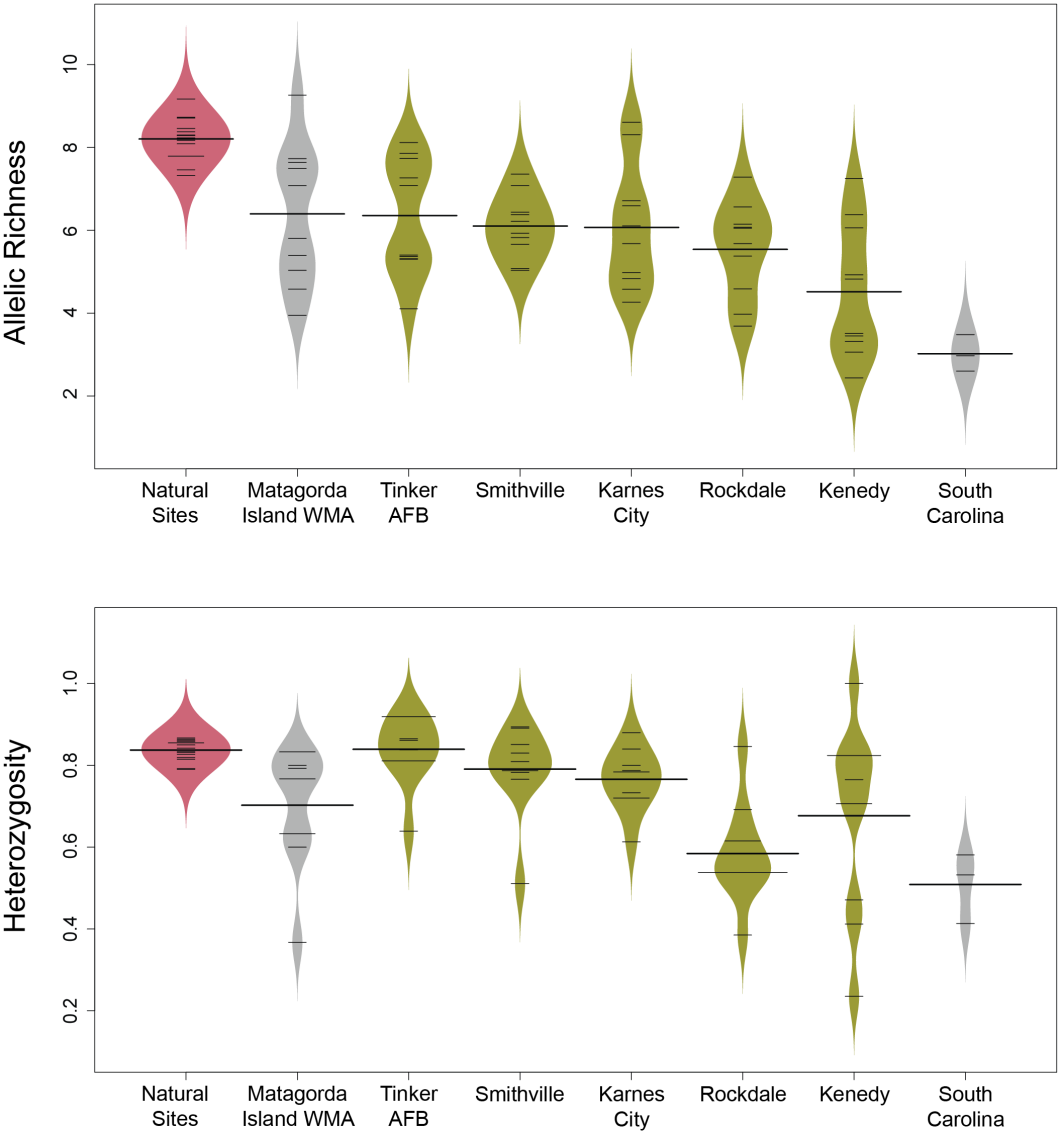
Violin plots for average allelic richness (top panel) and observed heterozygosity (bottom panel) (*n* = 10 microsatellite loci) of Texas horned lizards (*Phrynosoma cornutum*) in five towns (green) compared to barrier island populations (Matagorda WMA) and South Carolina (data from Heuring et al., 2019) (gray), and the average of 15 natural sites (red) (data from Williams et al., 2019).

Allelic richness and observed heterozygosity were 30% and 13% lower respectively in the towns than in the 15 natural sites (*p* = .001 in both cases) (Figure 3). Allelic richness and observed heterozygosity in the towns were similar to Matagorda Island WMA and higher than found in the introduced population in South Carolina (Figure 3). Differentiation among the towns (F_ST_ = 0.144) was higher than among the 15 natural sites (F_ST_ = 0.038, *p* = .002). Standardized F'_ST_ values between the towns were higher, with a global F'_ST_ of 0.636 and pairwise values ranging from 0.479–0.734, while between the 15 natural sites global F'_ST_ was 0.263 and pairwise values ranged from 0.017–0.665 (Table S1). The towns were strongly differentiated from the 15 natural sites (mean pairwise F'_ST_ between towns and natural sites = 0.598 ± 0.014 SE, range 0.317–0.856). Differentiation between Matagorda Island WMA and the 15 natural sites was similar to that seen with the towns (mean pairwise F'_ST_ between Matagorda Island WMA and natural sites = 0.524 ± 0.02 SE, range 0.390–0.664).

Average mitochondrial haplotype diversity was moderate for the towns (h = 0.48 ± 0.13) compared to the 15 natural sites (h = 0.67 ± 0.06) although the difference was not significant (Mann–Whitney W = 36, *p* = .16) (Table 1). Both Rockdale and TAFB had relatively high haplotype diversity. Population subdivision between the towns was higher for the control region (ɸPT = 0.389, AMOVA, *p* = .001) than the microsatellite loci (ɸPT = 0.246, AMOVA, *p* = .001). Of the 14 haplotypes detected in these towns, seven were shared with other sites in Texas (GenBank accession numbers MK100616, MK100617, MK100607, MK100621, MK100619, MK100625, MK100638) and seven were unique to the towns (2 in Smithville, 1 in Rockdale, 1 in Kenedy, and 3 at TAFB) (GenBank accession numbers OR843053‐OR843059). All 14 haplotypes clustered with haplotypes found in the eastern plains (Figure S1).

### 3.2 Bottlenecks and effective population size

There was a significant heterozygote excess relative to equilibrium expectations for TAFB, Smithville, and Karnes City suggesting they had experienced bottlenecks (*p* = .001–0.009). The Ne of the towns was low and averaged 27.1 ± 10.7 and ranged from 2.7–55.5 (Table 2). Matagorda Island WMA and the introduced population in South Carolina had similar Ne values to the towns (Ne = 24.6 and 16.9, respectively). Most (13 of 15) of the natural sites had confidence intervals and point estimates that included infinity (Table 2).

**TABLE 2:** Effective population size (Ne) with upper and lower 95% confidence limits (UCI and LCI) for Texas horned lizards (*Phrynosoma cornutum*) in four Texas towns and Tinker Air Force Base, Oklahoma and for 16 populations living in natural areas (data from Williams et al., 2019) and an introduced population in South Carolina (data from Heuring et al., 2019).

Site	*N*	Ne	LCI	UCI
Smithville	47	55.5	32.1	129.8
Rockdale	13	2.7	1.5	10.5
Tinker AFB	37	29.8	19.1	52.2
Karnes City	75	44.6	30.0	72.0
Kenedy	17	2.9	1.5	12.1
Matagorda Island WMA	30	24.6	14.8	49.6
South Carolina	128	16.9	12.5	22.8
Brewster Co.	31	63.4	35.5	184.3
Hueco Tanks SP	12	13.0	7.5	26.6
Seminole Canyon SP	17	41.5	10.6	∞
Midland Co.	30	440.9	103.9	∞
Yoakum Dunes WMA	36	300.2	92.0	∞
Matador WMA	55	511.4	155.8	∞
RPQRR	79	∞	560.5	∞
CMA	20	898.3	66.6	∞
East New Mexico	18	∞	208.2	∞
S.E. Colorado	13	156.5	23.5	∞
Camp Bowie	11	12.6	2.7	∞
Gray Co.	11	∞	65.8	∞
Mitchell Co.	14	346.4	20.6	∞
Chaparral WMA	63	∞	669.0	∞
Starr Co.	10	∞	146.1	∞

*Note*: *N* is the sample size for each population.

### 3.3 Anthropogenic movement of Texas horned lizards

**FIGURE 4:**
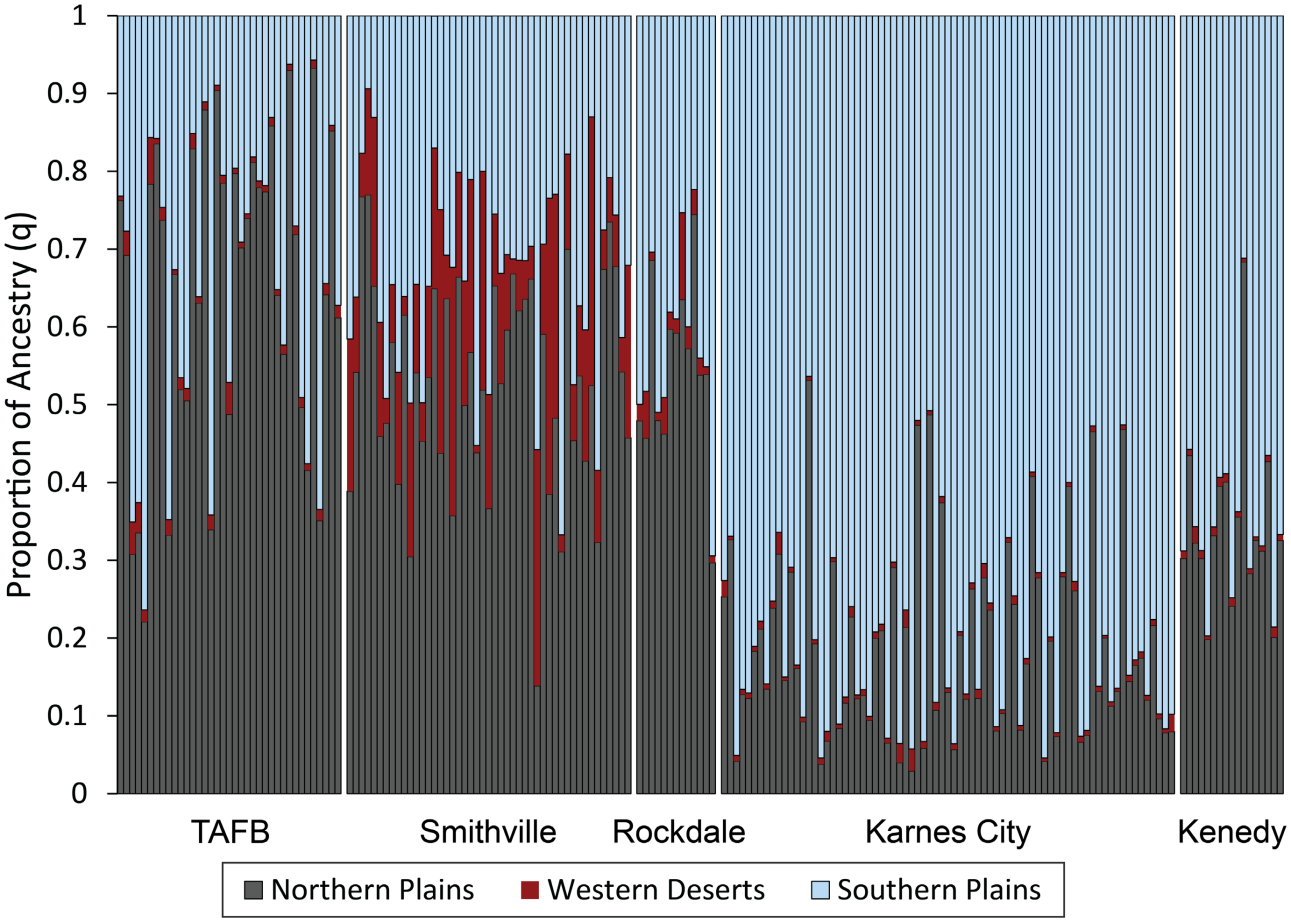
Assignment of Texas horned lizards (*Phrynosoma cornutum*) in Tinker Air Force Base (TAFB) in Midwest City, OK and four Texas towns to three genetic clusters. Each vertical line in the five panels indicates the proportion of ancestry (q) for an individual lizard with the colors representing the cluster or population assigned with STRUCTURE (Northern Plains, Western Deserts, Southern Plains).

There was a general trend of decreasing northern plains ancestry from TAFB (the most northern site) to Kenedy (the most southern site) (Figure 4). Few individuals (11% of 189 individuals) from towns had high ancestry values (q > 0.90) for a given cluster. If we use a cutoff value of q > 0.50 for assigning individuals to a cluster then most individuals (76% of 37) in TAFB were assigned to the northern plains, while 19% were assigned to the southern plains, and 5% were more evenly admixed between the northern and southern plains. In Smithville, most individuals (59% of 47 individuals) were assigned to the northern plains, 9% to the southern plains, and 32% were admixed between the northern plains, western deserts and southern plains. In Rockdale, most individuals (62% of 13 individuals) were assigned to the northern plains, 23% to the southern plains, and 15% that were evenly admixed between the northern, and southern plains. Most individuals (98% of 92 individuals) in Kenedy and Karnes City were assigned to the southern plains, while 2% were assigned to the northern plains.

### 3.4 Home range use in Kenedy and Karnes City

In Kenedy and Karnes City, the average home range size was 0.24 ha using 95% MCP and 0.39 ha using 100% MCP (Figure S2). Males (0.24 ± 0.10 ha using 95%, 0.33 ± 0.13 ha using 100% MCP) and females (0.24 ± 0.09 ha using 95% and 0.44 ± 0.21 ha using 100% MCP) did not differ significantly in their home range size (t_9_ = 0.04, *N* = 11, *p* = .97 using 95% MCP, and t_8_ = −0.46, *N* = 11, *p* = .66 using 100% MCP). About half (55%) of the lizards had home ranges that did not cross a road and were entirely within a street block (blocks were ~ 86 × 100 m) (Figure S2). The majority (98%) of all telemetry points (*N* = 421) for lizard relocations were within the street block that surrounded a horned lizard's home range and only 2% were located across a street in an adjacent town block. Three lizards were found in an adjacent town block (i.e., they moved across a road) once, one lizard twice, and one lizard five times. In all cases, these were small roads in residential neighborhoods with very low traffic volumes.

### 3.5 Karnes City spatial genetic structure

**FIGURE 5:**
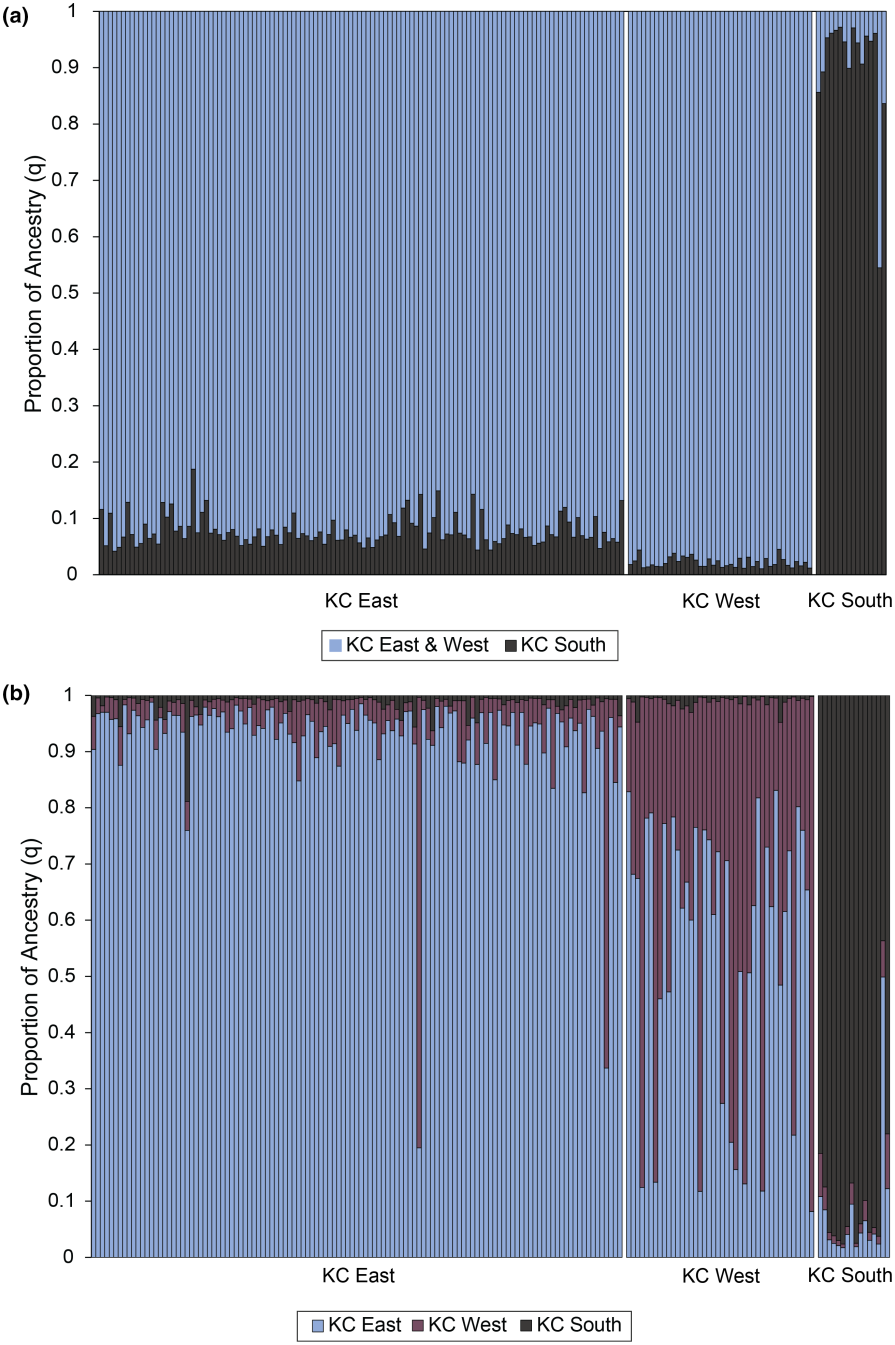
Bayesian clustering of multilocus genotypes from 177 Texas horned lizards, *Phrynosoma cornutum*, in three regions (south, east, and west) of Karnes City, Texas using STRUCTURE for (a) *K* = 2 and (b) *K* = 3. Each vertical line in the three panels indicates the proportion of ancestry (q) for an individual lizard with the colors representing the cluster or population identified in STRUCTURE.

**FIGURE 6:**
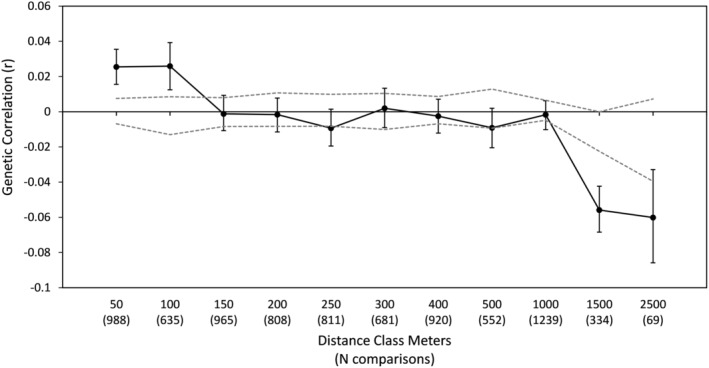
Correlogram showing genetic spatial autocorrelation (r) (solid line) for Texas horned lizards, *Phrynosoma cornutum*, in Karnes City, TX. Dotted lines are 95% confidence limits around 0 and error bars are 95% bootstrap confidence limits around r. There are significantly positive correlations at the 50‐ and 100‐meter categories and significantly negative correlations at 1500 and 2500 meters. Numbers in parentheses are the number of pair‐wise comparisons for each distance category.

There was significant subdivision within Karnes City between the west, east, and southern parts of town. Between east and west F'_ST_ = 0.064, between east and south F'_ST_ = 0.298, and between west and south F'_ST_ = 0.337 (*p* = .001 for all comparisons). The most likely number of clusters in Karnes City is *K* = 2, which splits the southern part of the city from the east and west subpopulations (Figure 5a). At *K* = 3, the southern cluster is still distinct and there is an indication of a third cluster in the western part of town. Most individuals (98% of 119 individuals) found in Karnes City East were assigned to the Karnes City East cluster, and two were assigned to the Karnes City West cluster. In Karnes City West, almost half (48% of 42 individuals) were assigned to the Karnes City East cluster and 12 were assigned to the Karnes City West cluster (Figure 5b). There was evidence for short‐distance dispersal with significant positive spatial genetic autocorrelation at 50 and 100 meters (Figure 6). Individuals within a cluster that were at the furthest distance classes (1000 and 1500 m) were significantly less similar than expected.

**FIGURE 7:**
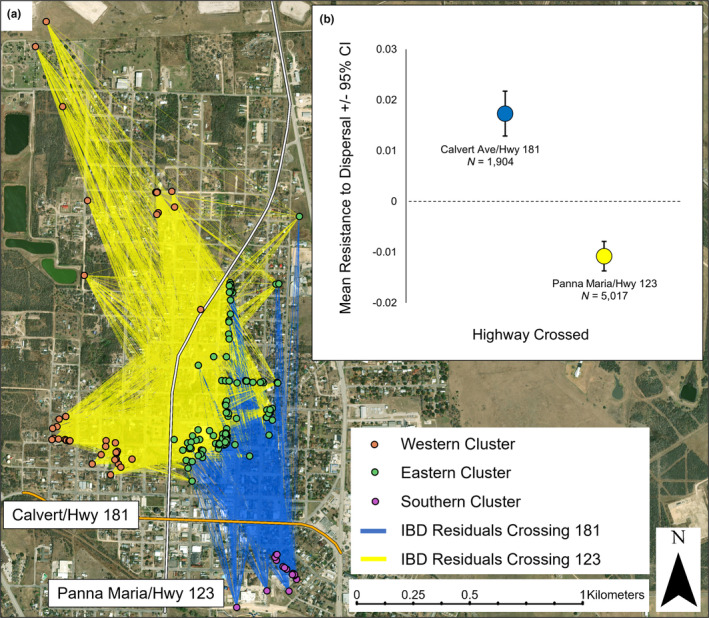
(a) The location of all 177 unique individual Texas horned lizards and line segments representing isolation by distance (IBD) residuals generated by ResDisMapper for residuals crossing highways. The southern cluster of individuals occurred south of Calvert Avenue/Hwy 181. The eastern cluster occurred north of Calvert Avenue/Hwy 181 and east of Panna Maria Avenue/Hwy 123 and the western cluster occurred west of Panna Maria Avenue/Hwy 123. (b) Residuals that crossed Calvert Avenue/Hwy 181 resisted dispersal (mean resistance to dispersal 0.017, 95% CI ± 0.004, *N* = 1904; yearly mean traffic volume 3829, TxDOT TPP Statewide Annual Average Daily Traffic), while residuals that crossed Panna Maria Avenue/Hwy 123 facilitated dispersal (mean resistance to dispersal −0.011, 95% CI ± 0.002, *N* = 5017; yearly mean traffic volume 1998, TxDOT TPP Statewide Annual Average Daily Traffic).

ResDisMapper used IBD residuals to map resistance to dispersal across Karnes City in 465 one ha grid cells (Figure 2a). There was statistical certainty that 80 grid cells were associated with resistance to dispersal and 113 grid cells were associated with facilitating dispersal. The remaining 272 cells did not deviate from the IBD trend with statistical certainty. The western side of town has many cells that facilitate dispersal as do many of the cells located along the edges of town where urbanization is less intense. The eastern and southern parts of town have more development, and there are continuous blocks of cells that resist dispersal in these areas. Cells that resisted dispersal contained significantly more impervious surface than cells that facilitated dispersal (Figure 2b). There was significant resistance to dispersal across Calvert Avenue/Hwy 181 and significant dispersal facilitation across Panna Maria Avenue/Hwy 123 (Figure 7a,b).

## 4 DISCUSSION

Few studies have been conducted on the genetic structure of small reptiles living within the urban matrix, and most of the studies have been conducted in much larger urban areas (e.g., Los Angeles, USA; Perth, Australia). Our results suggest that for species such as horned lizards, even very small towns can isolate populations. Texas horned lizards living in small towns have lower genetic diversity, higher differentiation, and smaller effective population sizes than populations located in more natural areas. The genetic diversity of these towns is similar to the diversity in a natural population living on a barrier island (Matagorda Island WMA). Island populations have lower genetic diversity and are more inbred than their rural counterparts due to isolation by an inhospitable surrounding matrix (Frankham, 1997). Three of the sites (TAFB, Rockdale, and Smithville) are surrounded by areas with no or low densities of Texas horned lizards and so probably have little opportunity for natural dispersal from outside of town to increase or maintain their levels of genetic diversity. Kenedy and Karnes City are in an area where Texas horned lizards still occur on the surrounding ranches, and yet these towns also have small effective population sizes and low genetic diversity. We suggest that physical structures such as roads, walls, and buildings serve to isolate horned lizards in these towns even when suitable surrounding habitat and populations exist.

### 4.1 Bottlenecks and effective population size

Allelic richness and heterozygosity were significantly lower in the towns compared to populations in natural areas suggesting they have experienced bottlenecks. Three of the towns (TAFB, Smithville, and Karnes City) had a significant heterozygote excess relative to equilibrium expectations, also suggesting they have experienced bottlenecks. Kenedy had a heterozygote excess across loci (7 of 10 loci had negative F_IS_ values) and very low genetic diversity, also suggesting this town has experienced a bottleneck. Rockdale had a heterozygote deficit across loci (9 of 10 loci had positive F_IS_ values) and very low genetic diversity suggesting the population is experiencing inbreeding.

The effective population sizes were very low in the towns and were lower than the recommended threshold to avoid inbreeding depression (Ne ≥100) (Frankham et al., 2014). These low population sizes may negatively affect these populations over the long term due to inbreeding and loss of longer‐term adaptability. Most of the natural sites had point values or confidence limits that included infinity indicating that there is no evidence for drift in those populations. At some sites, infinity values may have resulted from small sample sizes (*n* < 20) but in others for which sample sizes were larger it probably indicates that the populations are very large (>1000 individuals) which makes it difficult for the LD method to detect a signal of drift (Waples & Do, 2010). Some of the sites (Brewster Co., Hueco Tanks SP, Camp Bowie) had very low Ne point estimates like the towns, although these sites do not have the low microsatellite diversity observed in towns. This suggests these sites may have become small recently and there has not been enough time for drift to remove genetic variation.

Mitochondrial haplotype diversity was not significantly different between the towns and natural sites. Karnes City haplotype diversity (h = 0) was similar to barrier island populations (h = 0–0.067) but the other towns were more similar to some natural sites (Heuring et al., 2019; Williams et al., 2019). The low effective sizes and mtDNA haplotype diversity in some of the natural sites suggest that these sites are also isolated and starting to lose genetic variation, since it is expected that the signal of drift would first be noticed at mtDNA because of its matrilineal inheritance pattern and an effective population size that is one‐fourth that of nuclear genes (Birky Jr et al., 1983; DeSalle et al., 1987). The higher differentiation seen between towns at the mitochondrial locus than the microsatellite loci is also consistent with drift decreasing mtDNA diversity suggesting that the population structure we now observe has arisen relatively recently.

### 4.2 Anthropogenic movement of Texas horned lizards

If these towns have populations that are from the local area then we would expect to see a pattern of decreasing northern plains ancestry from Tinker AFB to Kenedy. This is the pattern that is observed; however, there was also evidence for a mix of ancestry from widely separated genetic clusters in the towns suggesting that some lizards were moved into town from other geographic localities. The ancestry values observed in towns contrasts with the average ancestry for individuals living in more natural areas of the northern cluster (~95% northern ancestry) and the southern cluster (~93% southern ancestry) (Williams et al., 2019). Tinker Air Force Base is located within the northern genetic cluster and yet had individuals with a high level of southern ancestry which is located over 600 km to the south. Most individuals in Smithville and Rockdale had a majority of northern ancestry but also more southern ancestry than TAFB. It is unclear, however, what the expectation would be for this region of Texas. Nevertheless, the presence of individuals with 20%–30% western ancestry in Smithville suggests lizards were probably also moved into that town at some point in the past, since the nearest area with western ancestry occurs over 600 km to the west. Kenedy and Karnes City are located within the southern cluster, and most individuals were assigned southern ancestry as expected. Several individuals in these towns had a majority of northern ancestry, which is located over 150 km to the north suggesting some lizards were also moved into the towns at some point in the past.

Texas horned lizards from both the northern plains and southern plains were introduced onto barrier islands in South Carolina in the mid‐1900s (Huering et al., 2019). This population has lower genetic diversity than the town populations. The higher genetic diversity of the towns than this introduced population and the general agreement between the ancestry in town and geographic location (northern ancestry decreases in towns from north to south) also suggest that populations like Smithville and Rockdale are not entirely due to introductions by residents. All of the towns appear to have some mixed ancestry suggesting lizards have been introduced in the past; however, these introductions have not been enough to offset the loss of genetic diversity.

### 4.3 Home range use in Kenedy and Karnes City

Lizard home ranges in the towns of Kenedy and Karnes City suggest that lizards are limiting most of their activity in the small patches of habitat that exist inside of town. Home ranges were predominantly located within a town block and were on the small end (0.25 ha) of 95% MCP home range sizes reported for this species which average ~ 1–2 ha in other areas of Texas (Anderson et al., 2017; Burrow et al., 2001, 2002; Fair & Henke, 1999). At TAFB, the 95% MCP home ranges (0.55–1.33 ha) (Wolf et al., 2013) were also larger than found in Kenedy and Karnes City possibly because they live in a relatively large, continuous habitat patch at TAFB that does not have impervious surfaces and that is isolated by the surrounding urban matrix rather than many smaller habitat patches that characterize Kenedy and Karnes City such as alleyways, yards, and small parks.

### 4.4 Karnes City spatial genetic structure

There was positive short‐distance genetic spatial autocorrelation in Karnes City which indicates limited short‐distance dispersal within town, and there was high differentiation  on either side of Calvert Avenue/Hwy 181 (F'_ST_ ≥ 0.30) and to a lesser extent across Panna Maria Avenue/Hwy 123 (F'_ST_ = 0.06). These levels of differentiation are high considering that F'_ST_ between natural areas separated by hundreds of kilometers was 0.263 (pairwise values ranged from 0.017–0.665). Resistance to dispersal was associated with areas of town that had more impervious surface which includes buildings, walls, and paved roads. Resistance to dispersal was especially pronounced across Calvert Avenue/Hwy 181 which is the main road through town, and which is also surrounded by the most development. Although there was significant but low differentiation across Panna Maria Avenue/Hwy 123, the IBD relationships suggest that dispersal is facilitated across this road when compared to the overall IBD trend for Karnes City.

Roads have been shown to impede movement of mammals (Epps et al., 2005; Golingay et al., 2013; Riley et al., 2006), birds (Fernández‐Juricic & Jokimäki, 2001), and reptiles (Brehme et al., 2012; Clark et al., 2010; Delaney et al., 2010; Hibbitts et al., 2017; Shepard et al., 2008) either through active avoidance or by inflicting mortality. The presence of suitable habitat along roads has been found to correlate with road mortality in some species, suggesting that animals are more likely to be near roads when suitable habitat is available (Grilo et al., 2009; Langen et al., 2009). Studies have also found that roads can vary in their permeability to movement. For instance, Brehme et al. (2012) found that dirt and secondary paved roads were penetrated by scrubland lizards (*Sceloporus occidentalis* and *Aspidoscelis hyperythra*) while rural highways were actively avoided, suggesting that lizards may avoid the noise, vibration, or visual disturbance produced by roads with steady traffic. It is not clear to what degree Texas horned lizards may avoid crossing roads with heavy traffic. Vehicular traffic is considered to be a significant source of mortality for Texas horned lizards in some areas (Montgomery & Mackessy, 2003; Sherbrook, 2002). Texas horned lizards often use the sides of unpaved and paved roads for thermoregulation and mate searching, and the easiest way to detect and capture these lizards in more rural areas is to drive slowly and look for them to move along the edges of the road (Fair & Henke, 1997; Henke, 2003; Henke & Montemayor, 1998; Sherbrook, 2002). The telemetry data indicated the lizards in town would travel across roads occasionally, but these were in residential areas with low traffic volumes and with suitable habitat. We never found lizards near Calvert Avenue/Hwy 181 but would occasionally find them next to Panna Maria Avenue/Hwy 123 which had lower traffic volumes, less development, and more suitable habitat along the road compared to Calvert Avenue/Hwy 181. The lower genetic differentiation and IBD relationships across Panna Maria Avenue/Hwy 123 suggest lizards can successfully move across this road and reproduce.

## 5 CONCLUSIONS

Smaller cities and towns can be important for conserving biodiversity since green space outside the city center is closer and potentially more connected to surrounding natural areas (Łopucki & Kitowski, 2017; Norton et al., 2016). Our data suggest that even a low level of urbanization can effectively isolate populations of some species, however, and so effective planning to maintain suitable habitat and corridors to facilitate movement will have to start at the earliest stages of urban development. The long‐term persistence of Texas horned lizards in these towns is closely tied to the availability of suitable habitat and prey, and they will disappear from an area if that habitat is altered or removed (Tucker et al., 2023; Wolf et al., 2013). Most of the sites where the lizards disappeared in Kenedy and Karnes City due to vegetation loss have not been recolonized even after suitable vegetation grew back, consistent with dispersal limitations in these towns (Tucker et al., 2023). As development in these towns increases, gene flow will be curtailed, leaving these populations even more isolated, small, and vulnerable to loss of genetic variation. If suitable habitat and corridors (such as undeveloped alleyways) that can facilitate movement between patches of habitat can be maintained within towns then the introduction of lizards from outside of town may help restore genetic variation and curtail inbreeding.

## AUTHOR CONTRIBUTIONS

**Ashley E. Wall:** Conceptualization (equal); formal analysis (equal); investigation (equal); methodology (equal); writing – original draft (equal); writing – review and editing (equal). **Daniella Biffi:** Formal analysis (equal); investigation (supporting); methodology (supporting); writing – original draft (equal); writing – review and editing (equal). **Alexis Ackel:** Investigation (equal); methodology (supporting); writing – review and editing (supporting). **Raymond W. Moody:** Conceptualization (supporting); investigation (supporting); writing – original draft (equal); writing – review and editing (equal). **Tom K. Stevens:** Formal analysis (equal); writing – original draft (equal); writing – review and editing (equal). **Dean A. Williams:** Conceptualization (equal); formal analysis (equal); funding acquisition (lead); investigation (equal); methodology (equal); writing – original draft (equal); writing – review and editing (equal).

## CONFLICT OF INTEREST STATEMENT

The authors declare no conflicts of interest.

## Supporting information


Appendix S1.

## Data Availability

Data for this study are available at: https://doi.org/10.18776/tcu/data/64953
