# Supplementary material for: Small towns limit dispersal and reduce genetic diversity in populations of Texas horned lizards
Source: Ecol Evol. 2024 Aug 6;14(8):e70112. doi: 10.1002/ece3.70112 (PMC11301276; doi:10.1002/ece3.70112)
Supplement: Supplementary file 1 — Appendix S1. [file ECE3-14-e70112-s001.docx]

Supplementary Material

Supplementary Table 1. Pairwise F’_ST_ (Standardized F_ST_ of Meirmans and Hedrick, 2011) between 5 populations of Texas horned lizards, *Phrynosoma cornutum*, in towns (SM – Smithville, RO – Rockdale, TAFB – Tinker Air Force Base, Ok, KC – Karnes City, KEN – Kenedy) and 16 natural areas (BR – Brewster Co., HT – Hueco Tanks SP, SC – Seminole Canyon SP, MD – Midland Co., YD – Yokum Dunes WMA, MA – Matador WMA, RPQRR – Rolling Plains Quail Research Ranch, CMA – Cross Bar MA, NM – East New Mexico, CO – S.E. Colorado, CB – Camp Bowie, GR – Grey Co., MI – Mitchell Co., CH – Chaparral WMA, MIL – Matagorda Island WMA, ST – Starr Co.) below diagonal, P- values are above diagonal. Data for natural area sites are from Williams et al. 2019.

| SM | RO | TAFB | KC | KEN | BR | HT | SC | MD | YD | MA | RPQRR | CMA | NM | CO | CB | GR | MI | CH | MIL | ST |  |
| --- | --- | --- | --- | --- | --- | --- | --- | --- | --- | --- | --- | --- | --- | --- | --- | --- | --- | --- | --- | --- | --- |
| **0.000** | 0.001 | 0.001 | 0.001 | 0.001 | 0.001 | 0.001 | 0.001 | 0.001 | 0.001 | 0.001 | 0.001 | 0.001 | 0.001 | 0.001 | 0.001 | 0.001 | 0.001 | 0.001 | 0.001 | 0.001 | SM |
| 0.708 | **0.000** | 0.001 | 0.001 | 0.001 | 0.001 | 0.001 | 0.001 | 0.001 | 0.001 | 0.001 | 0.001 | 0.001 | 0.001 | 0.001 | 0.001 | 0.001 | 0.001 | 0.001 | 0.001 | 0.001 | RO |
| 0.479 | 0.643 | **0.000** | 0.001 | 0.001 | 0.001 | 0.001 | 0.001 | 0.001 | 0.001 | 0.001 | 0.001 | 0.001 | 0.001 | 0.001 | 0.001 | 0.001 | 0.001 | 0.001 | 0.001 | 0.001 | TAFB |
| 0.666 | 0.611 | 0.601 | **0.000** | 0.001 | 0.001 | 0.001 | 0.001 | 0.001 | 0.001 | 0.001 | 0.001 | 0.001 | 0.001 | 0.001 | 0.001 | 0.001 | 0.001 | 0.001 | 0.001 | 0.001 | KC |
| 0.748 | 0.600 | 0.734 | 0.644 | **0.000** | 0.001 | 0.001 | 0.001 | 0.001 | 0.001 | 0.001 | 0.001 | 0.001 | 0.001 | 0.001 | 0.001 | 0.001 | 0.001 | 0.001 | 0.001 | 0.001 | KEN |
| 0.684 | 0.780 | 0.627 | 0.702 | 0.856 | **0.000** | 0.001 | 0.001 | 0.001 | 0.001 | 0.001 | 0.001 | 0.001 | 0.001 | 0.001 | 0.001 | 0.001 | 0.001 | 0.001 | 0.001 | 0.001 | BR |
| 0.596 | 0.758 | 0.663 | 0.722 | 0.849 | 0.394 | **0.000** | 0.001 | 0.001 | 0.001 | 0.001 | 0.001 | 0.001 | 0.001 | 0.001 | 0.001 | 0.001 | 0.001 | 0.001 | 0.001 | 0.001 | HT |
| 0.627 | 0.719 | 0.505 | 0.474 | 0.839 | 0.423 | 0.665 | **0.000** | 0.001 | 0.001 | 0.001 | 0.001 | 0.001 | 0.001 | 0.001 | 0.001 | 0.002 | 0.001 | 0.001 | 0.001 | 0.001 | SC |
| 0.577 | 0.629 | 0.458 | 0.512 | 0.670 | 0.277 | 0.602 | 0.186 | **0.000** | 0.152 | 0.002 | 0.009 | 0.101 | 0.038 | 0.001 | 0.001 | 0.302 | 0.055 | 0.001 | 0.001 | 0.001 | MI |
| 0.669 | 0.654 | 0.482 | 0.518 | 0.721 | 0.330 | 0.647 | 0.169 | 0.017 | **0.000** | 0.001 | 0.001 | 0.043 | 0.224 | 0.001 | 0.001 | 0.078 | 0.037 | 0.001 | 0.001 | 0.001 | YD |
| 0.537 | 0.623 | 0.378 | 0.520 | 0.679 | 0.368 | 0.553 | 0.238 | 0.069 | 0.091 | **0.000** | 0.001 | 0.001 | 0.001 | 0.001 | 0.001 | 0.037 | 0.012 | 0.001 | 0.001 | 0.001 | MA |
| 0.565 | 0.570 | 0.371 | 0.420 | 0.605 | 0.404 | 0.616 | 0.194 | 0.040 | 0.081 | 0.062 | **0.000** | 0.001 | 0.001 | 0.001 | 0.001 | 0.044 | 0.001 | 0.001 | 0.001 | 0.001 | RPQRR |
| 0.637 | 0.661 | 0.522 | 0.573 | 0.715 | 0.315 | 0.644 | 0.196 | 0.030 | 0.038 | 0.107 | 0.105 | **0.000** | 0.340 | 0.001 | 0.001 | 0.049 | 0.018 | 0.001 | 0.001 | 0.001 | CMA |
| 0.654 | 0.681 | 0.566 | 0.524 | 0.701 | 0.287 | 0.599 | 0.275 | 0.048 | 0.015 | 0.118 | 0.127 | 0.011 | **0.000** | 0.001 | 0.001 | 0.004 | 0.035 | 0.001 | 0.001 | 0.001 | NM |
| 0.525 | 0.705 | 0.550 | 0.553 | 0.745 | 0.415 | 0.584 | 0.269 | 0.199 | 0.224 | 0.267 | 0.281 | 0.225 | 0.219 | **0.000** | 0.002 | 0.027 | 0.001 | 0.001 | 0.001 | 0.001 | CO |
| 0.478 | 0.659 | 0.394 | 0.645 | 0.683 | 0.467 | 0.579 | 0.337 | 0.261 | 0.260 | 0.187 | 0.227 | 0.196 | 0.298 | 0.243 | **0.000** | 0.002 | 0.002 | 0.001 | 0.001 | 0.001 | CB |
| 0.494 | 0.730 | 0.334 | 0.452 | 0.759 | 0.362 | 0.634 | 0.128 | 0.018 | 0.051 | 0.069 | 0.057 | 0.069 | 0.120 | 0.106 | 0.193 | **0.000** | 0.007 | 0.001 | 0.001 | 0.001 | GR |
| 0.562 | 0.655 | 0.401 | 0.614 | 0.675 | 0.303 | 0.578 | 0.224 | 0.054 | 0.062 | 0.075 | 0.114 | 0.085 | 0.069 | 0.231 | 0.199 | 0.150 | **0.000** | 0.001 | 0.001 | 0.001 | MI |
| 0.514 | 0.600 | 0.400 | 0.317 | 0.631 | 0.600 | 0.602 | 0.361 | 0.369 | 0.391 | 0.310 | 0.308 | 0.445 | 0.443 | 0.453 | 0.432 | 0.325 | 0.359 | **0.000** | 0.001 | 0.001 | CH |
| 0.552 | 0.625 | 0.551 | 0.580 | 0.714 | 0.664 | 0.580 | 0.623 | 0.535 | 0.528 | 0.477 | 0.477 | 0.566 | 0.569 | 0.531 | 0.517 | 0.486 | 0.489 | 0.390 | **0.000** | 0.001 | MIL |
| 0.572 | 0.761 | 0.525 | 0.403 | 0.740 | 0.545 | 0.458 | 0.469 | 0.419 | 0.476 | 0.386 | 0.384 | 0.552 | 0.482 | 0.544 | 0.574 | 0.429 | 0.399 | 0.154 | 0.439 | **0.000** | ST |


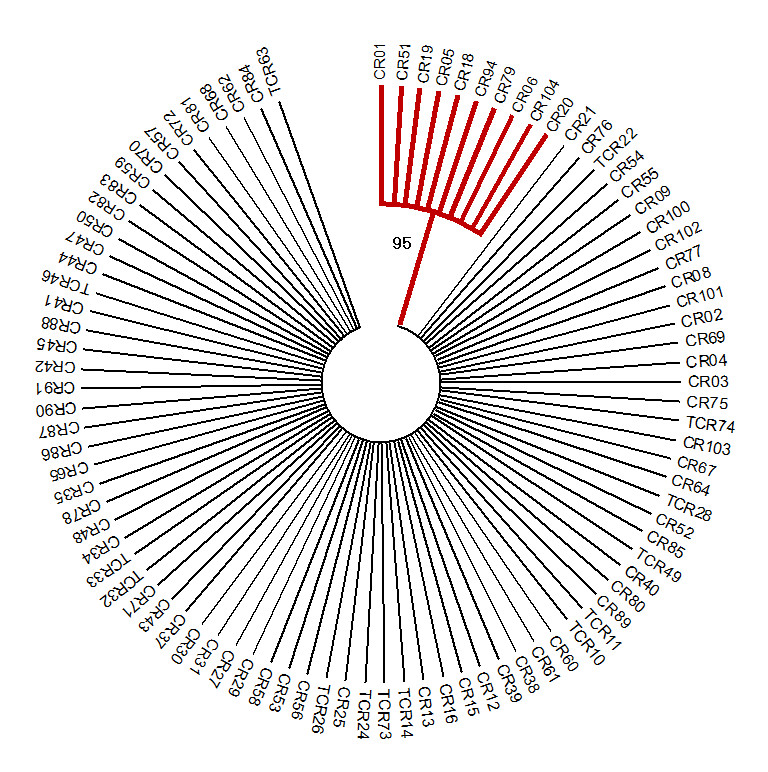


Supplementary Figure 1. Maximum likelihood tree for Texas horned lizard, *Phrynosoma cornutum*, mitochondrial control region (353 bp) haplotypes. Red lines are haplotypes in the western deserts clade and all others are in the eastern plains clade. Haplotypes named with a T are found within towns and are all part of the eastern clade. The model for nucleotide substitution is HKY + G + I as determined by the highest Bayesian information criterion (BIC) value. Maximum likelihood tree and BIC were calculated using MEGA 10 (Kumar et al. 2018).

Kumar S, Stecher G, Li M, Knyaz C, Tamura K. 2018. MEGA X: Molecular evolutionary genetics analysis across computing platforms. Molecular Biology and Evolution 35:1547-1549.


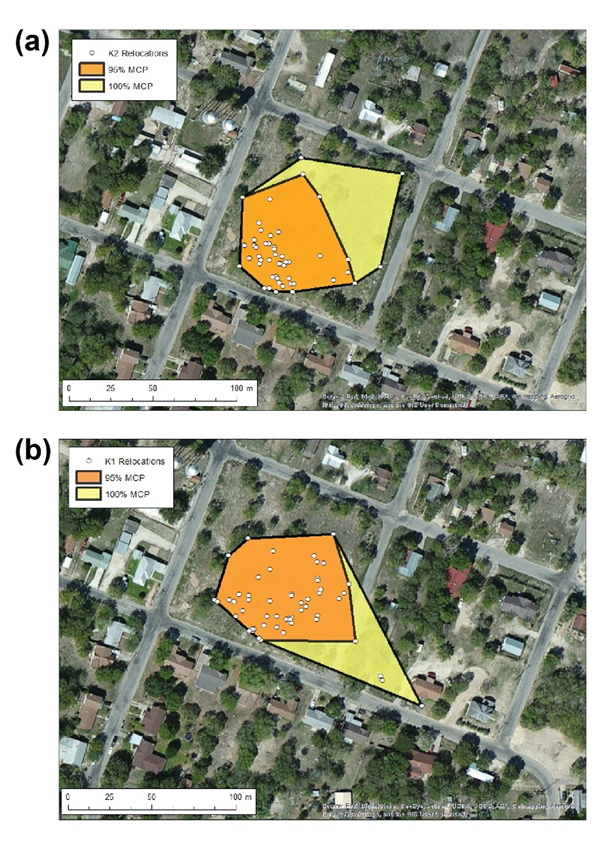


Supplementary Figure 2. Example of two Texas horned lizards, *Phrynosoma cornutum*, home ranges in Kenedy, Texas. The white points are lizard relocations, the orange polygon is the 95% minimum convex polygon (MCP) and the orange and yellow polygons are together the 100% MCP. The top panel shows a lizard whose home range is entirely within a street block and the bottom panel shows a lizard whose home range extends across a residential road into a neighboring street block.
